# In Metastatic Non-small cell Lung Cancer Platinum-Based Treated Patients, Herbal Treatment Improves the Quality of Life. A Prospective Randomized Controlled Clinical Trial

**DOI:** 10.3389/fphar.2017.00454

**Published:** 2017-07-17

**Authors:** Huiru Guo, Jia X. Liu, Hegen Li, Jan P. A. Baak

**Affiliations:** ^1^Department of Medical Oncology, Longhua Hospital, Shanghai University Shanghai, China; ^2^Division of Research, Dr. Med. Jan Baak AS Tananger, Norway; ^3^Division of Clinical Care Medical Practice, Dr. Med. Jan Baak AS Akersloot, Netherlands; ^4^Faculty of Sports and Health Sciences, Technical University of Munich Munich, Germany; ^5^Department of Pathology – Department for Molecular Biology, Stavanger University Hospital Stavanger, Norway

**Keywords:** lung cancer, non-small cell, metastatic, Quality of Life, Traditional Chinese Medicine, nausea, vomiting

## Abstract

**Background:** According to clinical experience, Traditional Chinese Medicine (TCM) herbs added to platinum-based therapy (PBT) improve the Quality of Life (QOL) in metastatic non-small cell lung cancer (NSCLC) patients, but this must be prospectively validated.

**Patients and Methods:** Based on clinical impressions regarding the effect of adding TCM herbs to platinum-based chemotherapy, we anticipated that 2 × 21 patients would be sufficient to obtain significant results with an α < 0.05 and power (1 - β) of 90%. To be on the safe side, we enrolled at least 28 patients in each group. In a prospective randomized controlled trial, 61 uniquely defined consecutive patients (PBT+PLACEBO, *N* = 32; PBT+TCM, *N* = 29) with stage IIIB-IV, Eastern Cooperative Oncology Group (ECOG) performance scores (PS) = 0–1 and TCM syndrome combined Qi-Yin deficiency were enrolled. These 61 patients were selected from originally 154 consecutive stage IIIB-IV lung cancer patients in the enrollment period. Patients were hospitalized and strictly controlled/surveyed during the entire 2-month treatment period, to guarantee use of or abstinence from TCM herbal and placebo fluids. Occurrence of nausea-vomiting, QOL by Functional Assessment of Cancer Therapy-Lung (FACT-L) scales and changes in ECOG “improved and stable rates” were compared before and after two treatment cycles.

**Results:** Before treatment, the clinico-pathologic and QOL features in PBT+PLACEBO and PBT+TCM patients did not differ (*P* > 0.10). The only side effects attributed by some of the patients to the TCM herbs were transient, mild gastric/abdominal heaviness in the first 2 weeks, but these also occurred amongst the PBT+PLACEBO patients (17 and 13%, *P* > 0.10). The incidence rates of nausea during treatment were 17% in PBT+TCM versus 75% in PBT+PLACEBO; vomiting rates were 14 and 56% (*P* < 0.0001 and 0.002). Moreover, ECOG “improved and stable rates” were 90% in the PBT+TCM versus 69% in the PBT+PLACEBO group (*P* = 0.04). In PBT+TCM patients, FACT-L social/familial and functional subscales were better after 2 months’ treatment (*P* = 0.02 and 0.03). Contrarily, in PBT+PLACEBO patients, the QOL variables total score, physical and emotional subscales were worse after PBT treatment (*P* = 0.03, 0.0001, and 0.003).

**Conclusion:** In stage IIIB-IV ECOG-PS = 0–1 NSCLC patients with Qi-Yin deficiency and platinum-based chemotherapy, adding TCM herbal medication improves the QOL. As this category of patients constitutes 40% of all metastatic NSCLCs, these results could have significant clinical impact.

## Introduction

Lung cancer is the leading cause of cancer-related mortality; about 1.4 million lung cancer patients die from their disease worldwide each year (American Cancer Society, 2011). In China, the mortality rate in 2008 was 30.8/100,000, making it the most lethal of all cancer types (http://www.moh.gov.cn/publicfiles/business/htmlfiles/zwgkzt/ptjnj/year2009/t-9.htm, 2009). More than 80% of lung cancers are histologically of the NSCLC type (Li, 2012). Moreover, most patients have distant metastases when diagnosed and have such a poor prognosis that surgery is not useful, leaving chemotherapy, radiotherapy and targeted therapy as the treatment options. The response rate of the standard first-line chemotherapy (platinum combined with third generation cytotoxic agents) of advanced lung cancers has improved significantly and currently MST is 8–10 months; the 1 year survival rate is 30% and higher (Li, 2012). Targeted medicines can significantly prolong survival, such as EGFR-targeting TKIs in EGFR mutant NSCLCs and crizotinib (a dual ALK/MET TKI) in ALK-positive NSCLC patients (Kwak et al., 2010; Camidge et al., 2011; Sebastian et al., 2014). On the other hand, in western countries such as Norway, the rate of EGFR mutations is much lower [i.e., 8% (Helland et al., 2011)] than in eastern Asia (40%). Moreover, at the moment targeted therapy is financially unaffordable for most patients in developing countries.

The global overall 5-year survival rate of NSCLC is only 16% (Jemal et al., 2010) and chemotherapy is notorious for its side effects. Many lung cancer patients with metastatic disease do not restrict themselves to conventional medicine, but seek additional help elsewhere. TCM herbal treatment has been reported to increase chemotherapy efficacy, reduce toxicity, prolong survival time, and strengthen immune functions (Liu et al., 1995; Chen, 1999; Zhu and Liu, 2000; Sun and Liu, 2001; Zhu et al., 2002; Sun and Liu, 2007a,b; Li et al., 2010). Chinese patients frequently use TCM herbs in addition to conventional chemotherapy, often without informing their treating specialists (Lam et al., 2009; Guo et al., 2011a).

We have recently shown in a case-control study, that adding TCM herbs to platinum-based chemotherapy in stage IIIB-IV pulmonary adenocarcinoma patients, significantly improves the 1 and 2 years’ survival rates (Guo et al., 2011b). Moreover, this improvement is not due to treatment delay (Guo et al., 2011a). Comparable results were obtained in independent studies in the United States on metastatic pulmonary and colorectal cancer (McCulloch et al., 2011a,b). The improved survival rates obtained in these three independent studies on patients with metastatic cancer from different organs are remarkably similar (Baak et al., 2011).

It is unlikely that these findings by various independent research groups from different countries are due to chance. However, prospective studies are required to finally support the survival advantage apparently caused by additional TCM herbal treatment. Moreover, before such studies will be approved by ethics committees, it is important to know if adding TCM herbal treatments has serious side effects and thus an effect on the QOL. Widespread clinical experience in China and elsewhere claims that using high quality TCM herbs has only minimal transient side effects and in fact improves the QOL in patients with metastatic lung cancer. However, as far as we know, a formal prospective RCT comparing the QOL specifically in patients with stage IIIB-IV pulmonary NSCLC treated with PBT, or PBT+TCM herbs, has not yet been undertaken. We therefore set out to study whether administering TCM herbal treatment to these patients has significant side effects and whether it improves the QOL.

## Materials and Methods

### Ethics and Patients

This randomized, prospective, controlled study on patients with first-time onset advanced NSCLC was approved by the Institutional Research Board of the Longhua University Hospital, Xuhui district, and the Respiratory University Hospital, Yangpu district, Shanghai, China before the study commenced. More recently, the Regional Ethics Committee of Health West (Bergen, Norway) also allowed one of the co-authors (JB) to participate in the study.

There were 154 consecutive new metastatic lung cancer patients between December 1, 2009 and February 1, 2011 at the Longhua University Hospital, Xuhui district, and the Respiratory University Hospital, Yangpu district, Shanghai, China. We used the following eligibility criteria: pathology-confirmed diagnosis of primary NSCLC (New edited diagnostic and treatment specification of common malignant tumors, 1997), inoperable stage IIIB-IV according to the UICC (Sobin et al., 2009), not pre-treated with chemotherapy, ECOG performance status < 2, 18 years of age or older, voluntary participation in the prospective study with signed informed consent to accept platinum-based chemotherapy plus randomized controlled use of either TCM herbs or no herbs fluids and TCM syndrome Combined Qi and Yin deficiency (for details of the latter, see below). **Table 1** shows the number of available, excluded and remaining included patients. In total, 64 patients fulfilled all criteria, but three refused chemotherapy. This left 61 patients who were randomized into PBT+PLACEBO (*N* = 32) or PBT+TCM group (*N* = 29). **Table 2** shows the characteristics of the patients. For details of the chemotherapy and TCM herbal treatment, see below.

**Table 1:** Total number of available, included and excluded patients.

Consecutive new patients with diagnosis metastatic lungcancer between December 1, 2009 until February 1, 2011,treated with TCM^∗^ herbal therapy		154 (100%)

**EXCLUDED**		**REMAINING**

Small cell cancer	31 (20.1%)	123 (79.9%)
No pathology	3 (1.9%)	120 (77.9%)
Performance score > 1	28 (18.2%)	92 (59.7%)
Pre-treated elsewhere	7 (4.5%)	85 (55.2%)
Refused chemotherapy	3 (1.9%)	82 (53.2%)
Other TCM^∗^ syndromes than Combined Qi and Yin deficiency	21 (13.6%)	61 (39.6%)


*^∗^TCM, Traditional Chinese Medicine.*

**Table 2:** Patients characteristics in the two treatment groups (PBT, Platinum-Based Chemotherapy; TCM, Traditional Chinese Medicine Herbal Treatment).

Characteristic		PBT+TCM (*N = 29*)	PBT+PLACEBO (*N = 32*)	Probability of no difference
Gender	Male	20 (20/29 = 69%)	28 (28/32 = 88%)	
	Female	9 (9/29 = 31%)	4 (4/32 = 13%)	0.15
Age (Years)	Range	38–77	31–77	
	Average	62	62	0.96
Smoking	No	11 (11/29 = 38%)	9 (9/32 = 28%)	
	Yes	18 (18/29 = 62%)	23 (23/32 = 72%)	0.29
Pathological subtype	Adenocarcinoma	20 (20/29 = 69%)	17 (17/32 = 53%)	
	Squamous carcinoma	5 (5/29 = 17%)	13 (13/32 = 41%)	
	Large cell carcinoma	1 (1/29 = 3%)	2 (2/32 = 6%)	
	Poorly differentiated carcinoma	3 (3/29 = 10%)	0 (0)	0.34
Stage	IIIB	8 (8/29 = 28%)	12 (12/32 = 38%)	
	IV	21 (21/29 = 72%)	20 (20/32 = 63%)	0.64
Metastatic organs	Brain	3 (3/29 = 10%)	3 (3/32 = 9%)	
	Lung	8 (8/29 = 28%)	9 (9/32 = 28%)	
	Liver	0 (0)	3 (3/32 = 9%)	
	Bone	8 (8/29 = 28%)	9 (9/32 = 28%)	
	Adrenal gland	1 (1/29 = 3%)	0 (0)	
	Other organs	9 (9/29 = 31%)	8 (8/32 = 25%)	0.23


Many Chinese patients receiving chemotherapy secretly use TCM herbs without informing their treating specialists (Lam et al., 2009; Guo et al., 2011a). On the other hand, adherence to using TCM herbs in non-supervised patients may not be always perfect. In order to carefully control and guarantee that the patients consistently used the placebo in the PBT+PLACEBO and TCM herbs in the PBT+TCM group, all patients were hospitalized throughout the study’s entire period of two treatment cycles (i.e., for 2 months), the administration and use of TCM herbal medication (PBT+TCM group) or placebo (in the PBT+PLACEBO group) was carefully controlled by medically trained personnel. Moreover, patients were kept under constant surveillance 24 h per day. It was therefore excluded as much as possible, that the PBT+PLACEBO patients secretly used TCM herbs obtained elsewhere and guaranteed that the PBT+TCM used their TCM herbs during the 2 months intervention and observation period.

### Chemotherapy and Pharmaceutical Intervention for Nausea and Vomiting

The patients were randomly treated with platinum-based chemotherapy according to one of the following regimens [there were no differences in outcome of lung cancer with these different platinum-based regimens (Schiller et al., 2002)]: (1) NP program (*N* = 19): vinorelbine 25 mg/m^2^, was administered on days 1, 8, and cisplatin 75 mg/m^2^, was administered on day 1 of a 4-week cycle. (2) TP program (*N* = 16): paclitaxel 135 mg/m^2^, and cisplatin 75 mg/m^2^ was administered on day 1. The cycle was repeated every 4 weeks. (3) GP program (*N* = 26): gemcitabine 1250 mg/m^2^, was administered on days 1 and 8, and cisplatin 75 mg/m^2^, was administered on day 1 of a 4-week cycle. Patients with grade 1–3 nausea or vomiting routinely received pharmaceutical anti-emetic Ondansetron (Zofran) treatment.

### TCM Qi and Yin Deficiency Syndrome

The patients were classified according to well-established criteria for the determination of TCM syndromes (Zheng, 2002; Shanghai Health Bureau, 2003). Only patients with the TCM syndrome criteria for both Qi and Yin deficiency were included, showing any of the following two main symptoms and two or more secondary symptoms.

*Main symptoms*: (1) Coughing with little sputum, (2) Shortness of breath, (3) Fatigue, and (4) Dry mouth.

*Secondary symptoms*: (1) Spontaneous sweating, (2) Night sweating, (3) Red tongue with teeth marks, and (4) Weak pulse.

### TCM Herbal Treatment

Traditional Chinese Medicine herbal treatment consisted of basic herbs which were given throughout the entire treatment period, and additional herbs which were selected each week based on specific symptoms the patients were experiencing during the treatment (in addition to the symptoms specific to the Qi and Yin deficiency syndrome mentioned above). **Table 3** gives a summary of the medications. The general TCM treatment principle was Benefiting Qi, Nourishing Yin and Removing Toxicity.

**Table 3:** Details of the herbs used and their indications.

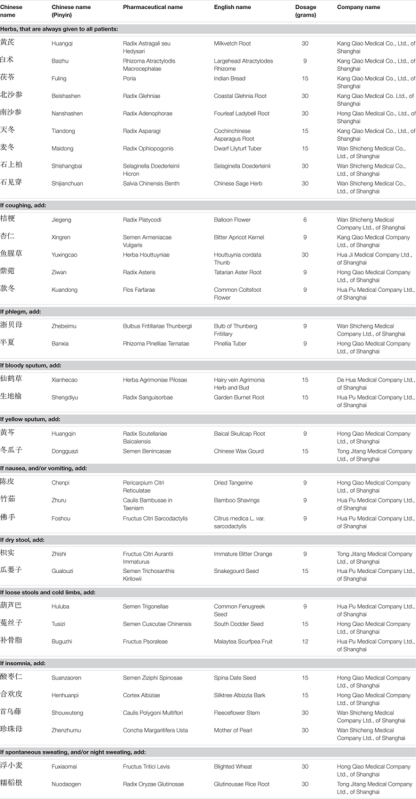

It is important to emphasize here, that the coding of the bottles with the different decoctions, were changed each week and only disclosed after the end of the study. Moreover, not only the patients were blinded to the contents of the decoctions, but also the doctors treating the patients and the nurses providing the decoctions to the patients each day.

The basic herbs were the following: Radix Astragali Mongholici 30 g, Rhizoma Atractylodis Macrocephalae 9 g, Poria 15 g, Radix Glehniae 30 g, Radix Adenophorae Strictae 30 g, Radix Asparagi 15 g, Radix Ophiopogonis 15 g, Selaginella Doederleinii Hieron 30 g, Salvia Chinensis Benth 30 g (**Table 3**).

Depending on specific symptoms, one or several of the following additional herbs were added:

If coughing: add Radix Platycodi 6 g, Semen Armeniacae Vulgaris 9 g, Herba Houttuyniae 30 g, Radix Asteris 9 g, and Flos Farfarae 9 g;
If phlegm: add Bulbus Fritillariae Thunbergii 9 g and Rhizoma Pinelliae Ternatae 9g;
If bloody sputum: add Herba Agrimoniae Pilosae 15 g and Radix Sanguisorbae 15 g;
If yellow sputum: add Radix Scutellariae Baicalensis 9 g and Semen Benincasae 15 g;
If nausea, and/or vomiting: add Pericarpium Citri Reticulatae 9 g, Caulis Bambusae in Taeniam 9 g, and Fructus Citri Sarcodactylis 9 g;
If dry stool: add Fructus Citri Aurantii Immaturus 9 g and Semen Trichosanthis Kirilowii 15 g;
If loose stools and cold limbs: add Semen Trigonellae 9 g and Semen Cuscutae Chinensis 15 g, and Fructus Psoraleae 12 g;
If insomnia: add Semen Ziziphi Spinosae 15 g, Cortex Albiziae 15 g, Caulis Polygoni Multiflori 30 g, and Concha Margaritifera Usta 30 g;
If spontaneous sweating, and/or night sweating: add Fructus Tritici Levis 30 g and Radix Oryzae Glutinosae 30 g.

### Placebo Decoction

In this randomized trial, the following considerations regarding the composition of the placebo decoction are important. On the one hand, the decoction should be as much neutral and without effects as possible. On the other hand, the patients should not know that the placebo drink is not the “real” TCM decoction used in lung cancer treatment. These conditions are not compatible with each other, as TCM herbal decoctions have a very specific color, smell, and taste. In China, this is the more impossible, as Chinese patients know very well these herbal decoctions’ characteristics due to the widespread use of TCM decoctions in Chinese families. Therefore, the Placebo should look, smell, and taste as a TCM herbal decoction, however, without the same strong effects as the “real” TCM herbal decoction. Another consideration is the fact, that this is a double-blinded trial. So, not only the patients, but also doctors and nurses do not and should not know which decoctions are “real” and which are “placebo”. On the other hand, doctors, nurses, and also patients participating in the study knew beforehand that there was a chance for patients to get a Placebo drink. In addition, they also had certain expectations from the effect of the “real TCM decoctions”. One of them is, that the side effects of chemotherapy are diminished by additional TCM herbal treatment. Therefore, if there would be too many side effects of chemotherapy in patients receiving Placebo drinks, the doctors and nurses could easily become suspicious and “betray” this by their body language to the patients. Even worse, if patients would get the feeling that they got “the wrong decoctions” they could consider withdrawing from the study. Consequently, to a certain degree we should meet these expectations by giving a Placebo with not only the typical smell, color, and taste, but also have some mild side effects of chemotherapy reducing effect.

It is well-known that nausea and vomiting are very frequent in platinum-based chemotherapy, as platin belongs to the highly emetogenic chemotherapy agents, with an incidence of nausea and vomiting of 90% and higher (CINV Risk factors, 2011; Protocol for the use of antiemetics to prevent chemotherapy-induced nausea, and vomiting, 2011). Nausea and vomiting are the most feared chemotherapy side effects. Up to 20% of patients receiving highly emetogenic agents postponed or even refused potentially curative treatments in the 1980s, before nausea and vomiting preventing pharmaceutical medicines were available (Gill et al., 2006). Finally, no doubt, nausea and vomiting strongly influence the other QOL features.

We therefore decided to choose herbs as Placebo, which gave the specific color, smell, and taste of TCM-herbal decoctions and have some Spleen Qi strengthening effect and thereby slightly diminish eventual nausea and vomiting. Based on clinical experience, the following herbs which have a Spleen qi strengthening effect amongst others, were chosen: Radix Codonopsis 9 g, Atractylodis Macrocephalae rhizoma 9 g, Poria 15 g, Pinelliae ternatae rhizome 9 g, Pericarpium citri reticulatae 9 g, and Glycyrrhizae radix 6 g.

It could not be avoided, that these herbs may have clinical effects. Atractylodes Macrocephalae rhizoma supports qi of the middle jiao, and can suppress nausea and vomiting, amongst others. Likewise, Poria drains and transforms dampness in the middle and lower jiao, reduce loss of appetite, abdominal distension and pressure and diarrhea. These two herbs had been used in our previous study, where TCM herbal treatment strongly improved prognosis (Guo et al., 2011b) and in fact also were in the PBT+TCM intervention treatment used in the current study. We realized that the current Placebo therefore was a compromise, as it partly could have some of the effects of the “true TCM decoctions” on the QOL features, but we regarded it as the best possibility.

### Preparation of the Decoctions

The herbal decoctions were supplied by the Pharmacy Department of the Longhua Hospital. The herbs, decoction and further preparation protocols and procedures are regularly controlled by the Chinese Food and Drug Administration (see below, in the section “Assessment of the Quality and Chemical Content of the TCM Herbs Used”). The herbal decoctions were all anonymized, and the codes of the different bottles were regularly changed during the study, to prevent bias amongst the patients, doctors, and nurses. The decoctions were administered orally twice per day (250 ml in the morning and 250 ml in the evening, 1 h after meals), for 2 months, under strict supervision by medically trained personnel, as described above in the section “Ethics and Patients”. We controlled the liver and kidney enzymes during the treatment cycles, but no major changes occurred in any of the patients.

### Assessment of the Quality and Chemical Content of the TCM Herbs Used

The herbs used were obtained from high quality companies, as shown in **Table 3**. All herbs used are well established and widely used as traditional Chinese medications and their active components have been described in detail in the Chinese Pharmacopoeia 2006, National Pharmacopoeia Commission, published by the China Medical Science and Technology Press, Beijing, China. Moreover, the production and quality control of the herbs, by the companies supplying the herbs to the Pharmacy Department of the Longhua University Hospital, is also very rigid and under strict control of the Chinese Food and Drug Administration. This implies, that all batches sold, are regularly controlled by the companies for toxic and microbiological impurities (pesticides, heavy metals, hormones, bacterial or fungal contamination), macroscopic and microscopic morphological analysis, and content of active chemical components, by fingerprinting and HPLC. We therefore did not perform during our study analysis of the active components of the decoctions delivered by the Pharmacy Department of the Longhua University Hospital. On the other hand, we did test later at the Norwegian Toxicology Institute, Oslo a representative decoction sample of the herbs from the Longhua Hospital Pharmacy Department. In agreement with the findings in China, we did not find detectable evidence for heavy metals, pesticides, or hormones. This confirms the quality of the herbs used in the present study.

### Assessment of Quality of Life

The American FACT-L Quality of Life Functional Assessment of Cancer Therapy-Lung version 4 (FACT-L v4) questionnaire and its lung cancer subscale (LCS) (Cella et al., 1993) were assessed at baseline, and on day 1, 28 of each treatment cycle period.

The ECOG score after the treatment was categorized as improved (score decreased ≥ 1), stable (score not changed) or worse (score increased ≥ 1).

Nausea and vomiting was assessed according to the National Cancer Institute’s Common Terminology Criteria for Adverse Events Nausea, Vomiting (National Cancer Institute, 2015), as follows: Nausea: grade 1, Loss of appetite without alteration in eating habits; grade 2: Oral intake decreased without significant weight loss, dehydration, or malnutrition; grade 3: Inadequate oral caloric or fluid intake; tube feeding, TPN, or hospitalization indicated; grade 4–5, Grade not assigned. Vomiting: grade 1: 1–2 episodes (separated by 5 min) in 24 h; grade 2: 3–5 episodes (separated by 5 min) in 24 h; grade 3: ≥6 episodes (separated by 5 min) in 24 h; tube feeding, TPN, or hospitalization indicated; grade 4: Life-threatening consequences; urgent intervention indicated; grade 5: Death.

We recorded the QOL items four times in each patient: before and after treatment of each cycle. As there were two cycles, totally four times. For the data analysis and comparison, we used only two scores: the one before the first cycle treatment (first month), and after the second cycle treatment (second month).

### Statistical Analysis

SPSS version 21 was used for the statistical analyses (SPSS, Chicago, IL, United States) and MedCalc (MedCalc Software, Mariakerke, Belgium) for power calculations and assessment of the sample size. Based on the previously published improvement of survival results and clinical impressions regarding the effect of adding TCM herbs to platinum-based chemotherapy, we anticipated that 2 × 21 patients would be sufficient to obtain significant results with an α < 0.05 and power (1 - β) of 90%. To be on the safe side, we enrolled at least 28 patients in each group. The paired-test was used to compare the continuous features. Frequency distribution with the chi-square test was used in numeration data. In all analyses, *P* < 0.05 was used as the level of significance. SPSS Statistics is a software package used for logical batched and non-batched statistical analysis. Long produced by SPSS Inc., it was acquired by IBM in 2009. The software name originally stood for SPSS, reflecting the original market, although the software is now popular in other fields as well, including the health sciences and marketing.

## Results

### Before Treatment

There were no differences (*P* > 0.13) in any of the characteristics between the two treatment groups, showing that there has not been any selection bias during randomization (**Table 2**).

### Side Effects of the TCM Herbs

As TCM drinks were given on the same day as the chemotherapy, the “additional side effects of TCM treatment” were difficult to assess. However, some patients in the PBT+TCM group complained of very mild gastric or abdominal heaviness in the first 2 weeks (in 5/29 = 17%), which they attributed to “the additional drinks,” but these complaints also occurred in 4/32 = 12% of the PBT++PLACEBO group patients, *P* > 0.10. These mild gastro-abdominal complaints in the first 2 weeks in the PBT+TCM and the PBT+PLACEBO groups were transient and none of the patients had to discontinue the use of TCM fluids. There were no significant changes in the liver and kidney enzymes during the treatment cycles.

### Comparison of QOL in Each of the Two Treatment Groups before and after Treatment

In PBT+PLACEBO patients, most QOL features showed a trend of being worse after PBT-treatment and the FACT-L physical and emotional subscales and the Total Score were significantly worse after than before treatment (0.0001, 0.003, and 0.03, respectively). On the other hand, the Social/Family subscale improved (**Table 4**).

**Table 4:** Quality of Life Changes of patients with advanced lung cancer (FACT-L)^∗^ (PBT = Platinum-Based Chemotherapy; TCM, Traditional Chinese Medicine Herbal Treatment).

Subscale	Group	*N*	Before treatment (Mean and standard error)	After treatment (Mean and standard error)	Probability of no difference
Physical	PBT+TCM	29	29.9 ± 0.65	29.1 ± 0.62	0.16
	PBT+PLACEBO	32	31.3 ± 0.63	28.6 ± 0.56	0.0001
Probability of no difference			0.12	0.54	
Social/Family	PBT+TCM	29	21.6 ± 0.65	22.6 ± 0.53	0.02
	PBT+PLACEBO	32	20.7 ± 0.58	21.8 ± 0.54	0.01
Probability of no difference			0.27	0.32	
					
Emotional	PBT+TCM	29	22.0 ± 0.69	22.3 ± 0.67	0.5
	PBT+PLACEBO	32	24.3 ± 0.67	21.9 ± 0.68	0.003
Probability of no difference			0.61	0.65	
Functional	PBT+TCM	29	9.5 ± 1.00	11.1 ± 0.68	0.03
	PBT+PLACEBO	32	9.8 ± 0.82	9.2 ± 0.54	0.56
Probability of no difference			0.84	0.03	
Lung cancer subscale	PBT+TCM	29	30.5 ± 1.26	31.8 ± 0.64	0.21
	PBT+PLACEBO	32	32.4 ± 0.44	31.3 ± 0.53	0.08
Probability of no difference			0.14	0.59	
Total score	PBT+TCM	29	114.5 ± 2.44	116.7 ± 2.13	0.29
	PBT+PLACEBO	32	118.1 ± 1.54	112.9 ± 1.77	0.03
Probability of no difference			0.2	0.17	


*^∗^FACT-L, Functional Assessment of Cancer Therapy-Lung.*

In the PBT+TCM group, the Social/Family, and functional subscales improved following treatment than prior to treatment commencement (*P* = 0.02 and 0.03). On the other hand, none of the other subscales became worse (**Table 4**).

### Comparison of QOL in Each of the Two Treatment Groups after Treatment

The incidence rates of nausea during treatment were 17% in the PBT+TCM versus 75% in the PBT+PLACEBO group (**Table 5**). Likewise, vomiting rates were 14 and 56% (*P* < 0.0001, 0.002). Moreover, nausea and vomiting were not only less frequent in the PBT+TCM group than in the PBT group, but also less severe (*P* = 0.002, **Table 5**). Consequently, PBT+TCM patients received ondansetron much less frequently than the PBT+PLACEBO group. The evaluation of the incidence and degree of severity of nausea and vomiting was performed at the end of the second treatment month, after the second chemotherapy cycle. This means, that all patients with grades 1–3 nausea and vomiting already had received the anti-emeticum ondansetron, if they required it. Moreover, patients in the PBT-TCM intervention group had received ondansetron much less often than those in the PBT+PLACEBO group. In spite of this, nausea and vomiting was much less frequent and severe in the PBT+TCM group. This proves, that adding TCM to PBT treatment strongly reduces nausea and vomiting and such patients require much less ondansetron treatment.

**Table 5:** Nausea and vomiting in the two treatment groups (PBT, Platinum-Based Chemotherapy; TCM, Traditional Chinese Medicine Herbal Treatment; N, Number of Patients).

Group	*N*	Grade 1	Grade 2	Grade 3	Grade 4	Grade 5	Rate (%)	Probability of no difference
**Nausea**								
PBT+TCM	29	5	0	0	0	0	17	*P* < 0.0001
PBT+PLACEBO	32	15	9	0	0	0	75	
**Vomiting**								
PBT+TCM	29	4	0	0	0		14	*P* = 0.002
PBT+PLACEBO	32	12	6	0	0	0	56	


Moreover, ECOG “improved and stable rates” were 90% in the PBT+TCM versus 69% in the PBT group (*P* = 0.04).

In agreement with this, the Functional subscale demonstrated improved results in the PBT+TCM group before and after treatment (*P* = 0.03), but not in the PBT+PLACEBO group (*P* = 0.56). Moreover, the differences after treatment were also significantly better in the PBT+TCM group compared to the PBT+PLACEBO group (*P* = 0.03, **Table 4**).

## Discussion

As far as known, this is the first Quality of Life RCT research and TCM herbals in which the patients were hospitalized and under 24-h surveillance during the whole 2 months’ treatment period. This is of the utmost importance, to control the circumstances/social environment and to eliminate patient’s bias (like taking extra herbals or vitamins without noticing the treating physicians). Moreover, one could object, that the number of patients is relatively small. However, the power calculations (based on the clinical empirical expectations from the senior TCM specialists who are co-authors of the current study), before the study started, made clear that the number of patients enrolled should be enough. If not, we would have enrolled more patients. Most important, the patients group studied is very pure (NSCLC only, Stage and performance score 0–1, TCM syndrome unique, all received platinum-based chemotherapy, all patients hospitalized and supervised 24 h per day during the observation period, TCM herbal decoctions administered by nurses). In fact, the total group came from a much larger group of 154 consecutive stage IIIb-IV lung cancer patients. It is the strict definition of our selection criteria, which reduces the patient numbers, but makes this a very pure patient group, which makes the study unique.

In this tightly controlled prospective RCT in patients with stage IIIB-IV NSCLC and ECOG performance score 0–1 in addition to the TCM syndrome Combined Qi and Yin deficiency at the time of diagnosis, adding TCM herbs during the first two cycles of platinum-based chemotherapy improves nausea and vomiting, ECOG rate and the QOL. Such patients constituted 40% of all patients with metastatic lung cancer presenting at our hospital. Patients with other characteristics exhibit very different clinical symptoms, clinical behavior and outcome.

Apart from the Social/Family feature, all QOL features point in the same direction; QOL in the PBT group was reduced after than before treatment but improved in the PBT+TCM group. Moreover, after treatment, the QOL features in the PBT+TCM group all showed a better trend than those in the PBT group.

The high incidence and the very low overall survival rate for advanced (stage IIIB and IV) lung cancer treated with cytostatic drugs, are strong motivators for many patients to seek new therapeutic avenues which may improve prognosis and QOL. In the past, patient well-being often has been regarded as less important than survival improvement and not tangible enough for straight forward scientific assessment. Over the past decade, however, reducing the suffering of cancer patients has become increasingly regarded as an important therapeutic goal and a well-established end-point in clinical trials, especially when survival improvement is marginal. Medical oncologists today are expected to seriously discuss with the patients the QOL as a trade-off and end-point in treating advanced NSCLC. Moreover, QOL is an independent prognostic factor for patients with NSCLC (Kosmidis, 1996).

In a previous study, we concluded that adding TCM herbal medication to platinum-based chemotherapy in advanced lung cancer patients could prolong survival time, but QOL was not evaluated. Good QOL is important as prolonged survival time with good QOL meets the principle “to live with a malignant tumor” (Sun et al., 2012). TCM considers not only overall survival, but also can increase chemotherapy efficacy, reduce toxicity, and strengthen immune functions (Liu et al., 1995; Chen, 1999; Zhu and Liu, 2000; Sun and Liu, 2001, 2007a,b; Zhu et al., 2002; Li et al., 2010). In the current study, we have found evidence supporting that adding TCM herbs also improves QOL. This confirms the long-term clinical experience of many TCM practitioners and patients.

A very important question is, how TCM herbs work on NSCLC, and the QOL features. In a previous paper, we have tried to summarize the possible prognosis-improving mechanisms (Baak et al., 2011). There can be direct (anti-proliferative, apoptosis-inducing, anti-invasive, anti-metastatic and diminish or block the capacity of metastasizing tumor cells to form distant colonies) and indirect effects on the tumor (by reducing tumor-favoring neuro-endocrine conditions and/or stimulating anti-tumor effects of the immune system).

Regarding fatigue, this also occurs in neurological diseases and chronic inflammatory diseases. Increasing evidence points to genetic and molecular mechanisms that are activated and cause fatigue during inflammation and cellular stress conditions, and signaled via neuro-immune and oxidative/nitrosative stress pathways. Heat shock proteins (HSP), particularly HSP90α, also may signal fatigue in chronic inflammation (Bårdsen et al., 2016), and possibly also in cancer and chemotherapy.

Nausea and vomiting are quite common after chemotherapy and are controlled by the brain. The most important cause of chemotherapy-induced nausea and vomiting is the activation of the chemoreceptor trigger zone by the chemotherapy agents circulating in the blood. Other pathways are also involved: from the brain cortex and limbic system (that reacts to sight, taste, smell, emotions, and pain), from a part of the ear that responds to motion (and so causes motion sickness in some people) and from some other organs and nerves (esophagus, stomach, and intestines). These signals are transmitted with the help of neurotransmitters. The sight and smell of chemotherapy are the main causes of ‘anticipatory nausea and vomiting.’ Antinausea drugs include prochlorperazine, droperidol, metoclopramide, and marijuana or marinol. The most common anti-emetic medications are serotonin receptor blockers, also known as 5-HT3 receptor antagonists such as Zofran (ondansetron, introduced in 1991) and Aloxi (palonosetron). Emend (aprepitant), a neurokinin receptor blocker, is another drug added to the 5HT3 blockers (see, for example American Cancer Society; National Comprehensive Cancer Network [NCCN], 2017). As far as we know, the effects of the herbs used in our study on these possible receptors, has not been studied. On the other hand, The Chinese herbal formula Xiao-Ban-Xia-Tang inhibits cisplatin-induced persistent eating of substances with no nutrition by down regulating obestatin in rats (Qian et al., 2011). Moreover, Gingerol has good activity against cisplatin-induced emesis in minks possibly by inhibiting central or peripheral increase of substance P and NK(1) receptors (Qian et al., 2010).

Analyses of the herbs used in our study, on these and other receptors and proteins would be a highly interesting future research topic.

An important question regards the functional mechanism by which TCM herbs prolong survival and improve the QOL of metastatic NSCLC patients. We have recently hypothesized the following possible mechanism (Baak et al., 2011). First, the TCM herbs have a proliferation-reducing effect on the tumor cells, in agreement with cell culture and animal studies (Baak et al., 2011). This anti-proliferative effect may constitute a direct toxic and apoptosis-inducing effect on the tumor cells, but also can potentially be mediated through TCM herbal effects on non-tumor factors such as estrogens or insulin. The second hypothetical important mechanistic effect is that due to the anti-proliferative effect, the cancer cells have a longer G0 phase and thereby can spend more time expressing MHC-differentiation surface antigens on their cell surface. This then in turn may give the patient’s immune system (which is stimulated by the TCM herbs), the opportunity to attack and kill the tumor cells.

Apparently, the most important QOL features improved under TCM herbal treatment (McCulloch et al., 2006) regard how the patients feel and function (“emotional” and “functional” FACT-L scales). As PBT+TCM patients received ondansetron much less frequently than the PBT+PLACEBO group, the favorable QOL effects in the PBT+TCM group are not due to more frequent administration of ondansetron (Zofran) in the intervention group.

Vomiting is strongly reduced by TCM herbs, the patients feel less tired and are more upbeat. This suggests that a factor causing fatigue is suppressed by TCM herbs. We have hypothesized that this suppressed factor could be an interleukin, for example IL-1or IL-6, as these have been implicated in fatigue in patients with immunologic diseases (Baak et al., 2011). It is interesting that the current results are in agreement with other studies (in breast cancer), showing that toxicity is significantly reduced when rationally chosen herbs are administered (Yaal-Hahoshen et al., 2011). This may be due in part to an effect on adaptive and innate immunity (Rachmut et al., 2013).

Strong points of the current study are the prospective randomized nature, the fact that all patients were hospitalized and surveilled 24 h per day during the 2 months treatment of the chemotherapy, to guarantee TCM herbal treatment adherence in the TCM group and exclusion of secret use of TCM herbs in the PBT group. Thirdly, although FACT-L QOL features are widely used and well reproducible (Wan et al., 2007), to guarantee the consistency of the QOL features as much as possible, all QOL criteria were assessed by the same investigator (HG), was very experienced when the study started and did not know whether the patients had received additional TCM herbal treatment. Finally, the patients all had NSCLC, stage IIIB and IV, eliminating heterogeneity in the stage and pathological subtypes of non-small cell and small-cell cancers. The patients were also homogeneous regarding ECOG performance scores 0–1 and the TCM syndrome, as all had deficiency of Qi and Yin. However, due to these very strict inclusion criteria, the number of patients was relatively small. Also, it was financially and socially not possible to hospitalize patients for longer than 2 months. The results therefore are limited to the 2 months (short-term) effects on QOL.

The current results encourage us to try to continue our attempts to organize TCM herbal adjuvant studies parallel to PBT treatment in advanced NSCLC patients outside of China. Such studies should preferably also include analyses of blood and tumor samples, to evaluate the molecular, biochemical, neuro-endocrine, and immunological features mentioned above, and possibly shed more light on the working mechanism of the TCM herbs.

## Conclusion

This RCT in inoperable (metastatic) stage IIIB-IV with ECOG 0-1 performance status NSCLC patients with the TCM syndrome of combined deficiency of Qi and Yin and treated with platinum-based chemotherapy, shows a significant better EORTC-QOL and ECOG Scores and much less Nausea/vomiting for patients receiving additional personalized TCM herbal medication, whilst having no significant side effects.

## Author Contributions

HG, JL, and HL: Design of the study, execution of the study, collecting data, analysis of data, writing the manuscript, and approval of the manuscript. JB: Design of the study, analysis of data, writing the manuscript, and approval of the manuscript.

## Conflict of Interest Statement

The authors declare that the research was conducted in the absence of any commercial or financial relationships that could be construed as a potential conflict of interest. The reviewer MH declared a shared affiliation, though no other collaboration, with one of the authors JB to the handling Editor, who ensured that the process nevertheless met the standards of a fair and objective review.

## Abbreviations

ALK: anaplastic lymphoma kinase factor
ECOG: Eastern Cooperative Oncology Group
EGFR: epidermal growth factor receptor
EORTC: European Organization for Research and Treatment of Cancer
FACT-L: Functional Assessment of Cancer Therapy-Lung, FACT-L 4.0
5-HT3 receptor: 5-hydroxytryptamine receptor
LCS: Lung cancer subscale of FACT-L
MET: mesenchymal-epithelial transition factor
MHC: major histocompatibility class
MST: median survival time
NK: natural killer cells
NSCLC: non-small cell lung cancer
P: probability of no significance
PBT: platinum-based therapy
Qi-Yin: Patients with the TCM syndrome deficiency of Qi and Yin
QOL: Quality of Life
RCT: randomized clinical trial
SPSS: Statistical Package for the Social Sciences
TCM: Traditional Chinese Medicine
TKI: tyrosine kinase inhibitor
TPN: total parenteral nutrition
UICC: international union against cancer

## References

[B1] American Cancer Society (2011). *Global Cancer Facts & Figures*, 2nd Edn Atlanta: American Cancer Society.

[B2] American Cancer Society. www.cancer.org/treatment

[B3] Baak J. P. Gyllenhaal C. Liu L. Guo H. Block K. I. (2011). Prognostic proof and possible therapeutic mechanisms of herbal medicine in patients with metastatic lung and colon cancer. *Integr. Cancer Ther.* 10 NP1–NP11 10.1177/1534735411421172 21948133

[B4] Bårdsen K. Nilsen M. M. Kvaløy J. T. Norheim K. B. Jonsson G. Omdal R. (2016). Heat shock proteins and chronic fatigue in primary Sjögren’s syndrome. *Innate Immun.* 162–167. 10.1177/1753425916633236 26921255 PMC4804286

[B5] Camidge D. R. Bang Y.-J. Kwak E. L. Shaw A. T. Lafrate A. J. Maki R. J. (2011). Progression-free survival (PFS) from a phase 1 study of crizotinib (PF-02341066) in patients with ALK-positive non-small cell lung cancer (NSCLC). *J. Clin. Oncol.* 29:2501 10.1200/jco.2011.29.15_suppl.2501

[B6] Cella D. F. Tulsky D. S. Gray G. Sarafian B. Linn E. Bonomi A. (1993). The functional assessment of cancer therapy scale: development and validation of the general measure. *J. Clin. Oncol.* 11 570–579. 10.1200/JCO.1993.11.3.570 8445433

[B7] Chen Z. (1999). Meta analysis on primary non-small cell lung carcinoma (NSCL) treated by TCM in China. *J. Trad. Chin. Med. Pharm.* 40 287–289.

[B8] CINV Risk factors (2011). *Cesamet Patient Education.* Somerset, NJ: Meda Pharmaceuticals.

[B9] Gill P. Grothey A. Loprinzi C. (2006). “Nausea and vomiting in the cancer patient,” in *Oncology, an Evidence-Based Approach*, eds Chang A. E. Hayes D. F. Pass H. I. Stone R. M. Ganz P. A. Kinsella T. J. (New York, NY: Springer), 1482–1496. 10.1007/0-387-31056-8_83

[B10] Guo H. Liu J. X. Xu L. Madebo T. Baak J. P. (2011b). Traditional Chinese medicine herbal treatment may have a relevant impact on the prognosis of patients with stage IV adenocarcinoma of the lung treated with platinum-based chemotherapy or combined targeted therapy and chemotherapy. *Integr. Cancer Ther.* 10 127–137. 10.1177/1534735410387599 21147812

[B11] Guo H. Liu L. Baak J. P. (2011a). Is the improvement of prognosis of patients with metastatic pulmonary adenocarcinoma treated with TCM herbal medicine due to lag time to treatment bias? *Integr. Cancer Ther.* 10 234–239. 10.1177/1534735411418112 21862517

[B12] Helland Å. Skaug H. M. Kleinberg L. Iversen M. L. Rud A. K. Fleischer T. (2011). EGFR gene alterations in a Norwegian cohort of lung cancer patients selected for surgery. *J. Thorac Oncol.* 6 947–950. 10.1097/JTO.0b013e31820db209 21623266

[B13] http://www.moh.gov.cn/publicfiles/business/htmlfiles/zwgkzt/ptjnj/year2009/t-9.htm. (2009). 2009 08.26.

[B14] Jemal A. Siegel R. Xu J. Ward E. (2010). Cancer statistics. *CA Cancer J. Clin.* 60 277–300. 10.3322/caac.20073 20610543

[B15] Kosmidis P. (1996). Quality of life as a new end point. *Chest* 109 110–112. 10.1378/chest.109.5_Supplement.110S 8635386

[B16] Kwak E. L. Bang Y. J. Camidge D. R. Shaw A. T. Solomon B. Maki R. G. (2010). Anaplastic lymphoma kinase inhibition in non-small-cell lung cancer. *New Engl. J. Med.* 363 1693–1703. 10.1056/NEJMoa1006448 20979469 PMC3014291

[B17] Lam Y. C. Cheng C. W. Peng H. Law C. K. Huang X. Bian Z. (2009). Cancer patients’ attitudes towards Chinese medicine: a Hong Kong survey. *Chin. Med.* 4:25 10.1186/1749-8546-4-25 PMC2805668 20042091

[B18] Li C. J. Sun J. Liu L. S. (2010). Regulatory effect of strengthening qi and nourishing yin recipe on expressions of ERα and CyclinD1 of Lewis lung cancer in C57 mice. *J. Trad. Chin. Med. Pharm.* 25 578–581.

[B19] Li J. L. (2012). Choice of treating advanced lung cancer. *Oncol. Prog.* 10 321–329.

[B20] Liu J. X. Shi Z. Xu Z. (1995). Studies on advanced primary adenocarcinoma of the lung treated by nourishing yin to replenish fluid and warming yang to benefit Qi. *J. Trad. Chin. Med. Pharm.* 36 155–158.

[B21] McCulloch M. Broffman M. van der Laan M. Hubbard A. Kushi L. Kramer A. (2011b). Lung cancer survival with herbal medicine and vitamins in a whole-systems approach: ten-year follow-up data analyzed with marginal structural models and propensity score methods. *Integr. Cancer Ther.* 10 260–279. 10.1177/1534735411406439 21824893

[B22] McCulloch M. Broffman M. van der Laan M. Hubbard A. Kushi L. Abrams D. I. (2011a). Colon cancer survival with herbal medicine & vitamins combined with standard therapy in a whole-systems approach: 10-year follow-up data analyzed with marginal structural models and propensity score methods. *Integr. Cancer Ther.* 10 240–259. 10.1177/1534735411406539 21964510 PMC4081504

[B23] McCulloch M. See C. Shu X. J. Broffman M. Kramer A. Fan W. Y. (2006). Astragalus-based Chinese herbs andplatinum-based chemotherapy for advanced non-small-cell lung cancer: meta-analysis of randomized trials. *J. Clin. Oncol.* 24 419–430. 10.1200/JCO.2005.03.6392 16421421

[B24] National Cancer Institute (2015). *Treatment-Related Nausea and Vomiting (PDQ^®^)–Health Professional Version.* Available at: http://www.cancer.gov/about-cancer/treatment/side-effects/nausea/nausea-hp-pdq 26389491

[B25] National Comprehensive Cancer Network [NCCN] (2017). *Preventing Nausea and Vomiting From Cancer Treatment.* www.nccn.org/patients/resources/life_with_cancer/managing_symptoms/preventing_nausea.aspx

[B26] New edited diagnostic and treatment specification of common malignant tumors. (1997). (Volume 9-Primary bronchogenic carcinoma), edited by Chinese Anti-Cancer Association, Beijing: Chinese Union Medical College Press, 737–781.

[B27] Protocol for the use of antiemetics to prevent chemotherapy-induced nausea and vomiting (2011). VHA pharmacy benefits management strategic healthcare group and medical advisory panel.

[B28] Qian Q. H. Chen W. Guo C. Wu W. Qian W. Li S. (2011). The Chinese herbal formula Xiao-Ban-Xia-Tang inhibits cisplatin-induced pica by down regulating obestatin in rats. *J. Ethnopharmacol.* 135 186–193. 10.1016/j.jep.2011.03.008 21396996

[B29] Qian Q. H. Yue W. Chen W. H. Yang Z. H. Liu Z. T. Wang Y. X. (2010). Effect of gingerol on substance P and NK1 receptor expression in a vomiting model of mink. *Chin Med J (Engl.)* 123 478–484. 20193490

[B30] Rachmut I. H. Samuels N. Melnick S. J. Ramachandran C. Sharabi Y. Pavlovsky A. (2013). Immunomodulatory effects of the botanical compound LCS101: implications for cancer treatment. *Onco Targets Ther.* 6 437–445. 23637542 10.2147/OTT.S42038 PMC3639221

[B31] Schiller J. H. Harrington D. Belani C. P. Langer C. Sandler A. Krook J. (2002). Comparison of four chemotherapy regimens for advanced non-small-cell lung cancer. *N. Engl. J. Med.* 346 92–98. 10.1056/NEJMoa011954 11784875

[B32] Sebastian M. Schmittel A. Reck M. (2014). First-line treatment of EGFR-mutated non-small cell lung cancer: critical review on study methodology. *Eur. Respir. Rev.* 23 92–105. 10.1183/09059180.00008413 24591666 PMC9487257

[B33] Shanghai Health Bureau (2003). “*Shanghai Traditional Chinese Medicine Syndrome Clinic Routine” Version 2*. Shanghai: Shanghai University of Traditional Chinese Medicine Press, 128–129.

[B34] Sobin L. H. Gospodarowicz M. K. Wittekind C. (2009). UICC “International union against cancer. Lung and pleural tumours,” in *TNM Classification of Malignant Tumours*, 7th Edn, eds Sobin L. H. Gospodarowicz M. K. Wittekind C. (Oxford: Wiley-Blackwell), 138–146.

[B35] Sun G. Liu J. X. (2001). Effect of Jin Fu Kang liquid on IL-10 and IFN in lung cancer patients. *Shandong J. Trad. Chin Med.* 20 721–722.

[B36] Sun G. Liu J. X. (2007a). Effect of Jin Fu Kang liquid on cytotoxic T lymphocytes in mice with Lewis pulmonary cancer. *Hunan J. Trad. Chin. Med.* 23 92–93.

[B37] Sun J. Liu J. X. (2007b). Effect of Jin Fu Kang Oral solution on expression of apoptosis related genes of human lung adenocarcinoma cells transplanted in nude mice. *Shanghai J. Trad. Chin. Med.* 41 69–71.

[B38] Sun Y. Liao M. L. Zhou Y. Z. (2012). *Lung Cancer. Version 3.* Shanghai: Shanghai Science and Technology Press, 522.

[B39] Wan C. Zhang C. Cai L. Tu X. Feng C. Luo J. (2007). Psychometric properties of the Chinese version of the FACT-L for measuring quality of life in patients with lung cancer. *Lung Cancer* 56 415–421. 10.1016/j.lungcan.2007.01.004 17316887

[B40] Yaal-Hahoshen N. Maimon Y. Siegelmann-Danieli N. Lev-Ari S. Ron I. G. Sperber F. (2011). A prospective, controlled study of the botanical compound mixture LCS101 for chemotherapy-induced hematological complications in breast cancer. *Oncologist* 16 1197–1202. 10.1634/theoncologist.2011-0150 21712486 PMC3228177

[B41] Zheng X. Y. (2002). *Guidelines of New Chinese Medicine Clinical Research (trial). Version 1.* Beijing: Chinese Medical Science and Technology Press, 216–221.

[B42] Zhu H. Liu J. X. (2000). Experimental study of “lung-benefiting and tumor-resisting drink” on neuroendocrine immunity in mice with Lewis pulmonary cancer. *Acta Univer. Trad. Med. Sin. Pharmacol. Shang.* 14 44–46.

[B43] Zhu Y. Gao H. Chen S. (2002). Study on the mechanism of YangyinQingfei prescription regulating immune function of mice with tumor. *Chin. Arch. Trad. Chin. Med.* 20 318–319.

